# Associations between digital health literacy and health inequalities among middle-aged and older adults: an urban–rural disparity perspective

**DOI:** 10.3389/fpubh.2026.1896811

**Published:** 2026-08-12

**Authors:** Jian Wu, Pingping An, Ning Chen

**Affiliations:** 1 College of Public Health, Zhengzhou University, Zhengzhou, China; 2 School of Politics and Public Administration, Zhengzhou University, Zhengzhou, China

**Keywords:** digital health literacy, health inequalities, health promotion, middle-aged and older adults, urban–rural disparity

## Abstract

**Background:**

In the digital age, digital health literacy has emerged as a critical factor influencing the physical and mental well-being of middle-aged and older adults. However, the relationship between digital health literacy and health inequalities remains underexplored. This study aims to investigate this association among middle-aged and older adults in urban and rural settings.

**Methods:**

Data were obtained from the 2021 Chinese General Social Survey (CGSS). The final analytical sample consisted of 1,672 respondents from the International Social Survey Programme (ISSP) Health Module after excluding individuals younger than 45 years and those with missing values on key variables. The digital health literacy index was constructed using the entropy weight method, which objectively determines indicator weights based on the relative information contained in each dimension. The linear probability model was employed to examine the relationship between digital health literacy and urban–rural health inequalities, given its straightforward coefficient interpretation and suitability for analyzing interaction effects.

**Results:**

Digital health literacy reduces urban–rural disparities in physical health among middle-aged and older adults by 0.2229 points [95% CI (−0.4329, −0.0129), *p* = 0.037] and in mental health by 0.2410 points [95% CI (−0.3908, −0.0913), *p* = 0.002], thereby enhancing overall well-being and narrowing health disparities. Heterogeneity analysis reveals that the effects of digital health literacy on reducing urban–rural disparities in physical health are stronger among married individuals, while its benefits for mental health inequalities are more evident among married individuals, those with lower educational attainment, and older adults. Mechanism analysis indicates that digital health literacy mitigates urban–rural health inequalities by promoting health-enhancing behaviors.

**Conclusion:**

Digital health literacy not only improves the physical and mental well-being of middle-aged and older adults but also effectively reduces urban–rural health inequalities.

## 1 Background

Urban–rural health disparities have garnered significant scholarly attention in both developed and developing nations (1–3). Health inequalities can result in severe consequences, including declines in social productivity, lower overall health levels, increased medical burdens, and the exacerbation of broader social inequalities. Consequently, mitigating these disparities is of paramount importance (4, 5). Over the past few decades, China has experienced rapid economic growth and social transformation. However, rural adults have consistently faced disadvantages in accessing medical and health services, as well as in overall health status (6). In China, these disparities are shaped not only by geographical differences but also by the hukou (household registration) system, an institutional arrangement that influences access to public services such as healthcare, education, and social welfare. Although the hukou system has undergone substantial reforms, differences in the allocation and accessibility of public resources between urban and rural hukou holders continue to contribute to unequal health opportunities. This disparity is particularly pronounced among middle-aged and older adults in rural areas, who have significantly more limited access to healthcare services and experience markedly poorer health conditions compared to their urban counterparts. According to the Fifth Sample Survey on the Living Conditions of China’s Urban and Rural Older Persons Datasets, the health status of rural middle-aged and older adults is considerably lower than that of their urban counterparts. Specifically, the proportion of rural older adults who rate their self-assessed health as “very good” or “fairly good” is 7.4 percentage points lower than that of urban older adults. Addressing urban–rural health disparities among middle-aged and older adults is not only essential for safeguarding their health rights and well-being but also a critical step toward promoting social equity and achieving sustainable, healthy development.

Previous studies have primarily focused on reducing urban–rural health disparities by integrating urban and rural medical insurance systems, expanding rural healthcare services, and addressing spatial disparities in medical resource distribution (5, 7). However, with the widespread adoption of the internet, health information dissemination and health management have increasingly transitioned to digital platforms, and digital health literacy has become an essential factor in improving adult health outcomes (8). Digital health literacy can be broadly defined as the ability to effectively search for, access, understand, evaluate, and utilize online health information to address or mitigate health-related issues (9). It is not only a crucial determinant of improved health outcomes for middle-aged and older adults but also a vital tool for managing chronic diseases and preventing health risks (10). Digital health literacy enhances health outcomes through several mechanisms, including improving access to health information by enabling individuals to acquire comprehensive health knowledge via the internet and enhance their overall understanding of health (11, 12); facilitating self-health management by allowing individuals to monitor physiological indicators using smart health tools, receive timely risk alerts, and undertake appropriate health interventions (13); supporting health-related decision-making by enhancing comprehension of medical reports and healthcare recommendations through digital platforms such as telemedicine consultations and online doctor evaluations (14); and promoting healthy behaviors by fostering the development of sustainable health habits through setting health goals, tracking personal health data, engaging in online health communities, and receiving personalized health guidance (15). A systematic review has demonstrated that digital health literacy is strongly associated with various health-related outcomes among older adults, including engagement in health-promoting behaviors, improvements in health-related quality of life, and better mental well-being (16). Furthermore, advancements in digital health literacy have the potential to overcome geographical barriers, presenting a novel opportunity to mitigate urban–rural health inequalities.

The impact of digital health literacy on urban–rural health inequalities is complex and multifaceted. On the one hand, digital health literacy can help mitigate health disparities arising from resource inequalities. Existing research indicates that rural adults can access professional health consultations and medical services through digital platforms, facilitated by the widespread adoption of digital health tools such as health management applications and telemedicine (17, 18). Online medical consultations enable rural middle-aged and older adults to obtain professional medical guidance efficiently and in a timely manner, thereby reducing healthcare access inequalities resulting from the uneven distribution of medical resources between urban and rural areas (6). A systematic review suggests that the utilization of smart devices and health applications can optimize the allocation and utilization of medical resources, thereby improving the efficiency of healthcare service delivery (19). Additionally, digital health technologies support rural adults in establishing online health-support networks (20). A survey-based study indicated that virtual interactions help compensate for deficiencies in rural health-support systems, thereby enhancing social participation and health awareness among rural adults (21). Later evidence found that digital health literacy exerts a significant positive impact on rural adults’ engagement in diverse health-related activities (22). Moreover, digital health technologies are characterized by their cost-effectiveness and inclusive nature, creating favorable conditions for reducing disparities in health management between urban and rural populations (6).

On the other hand, digital health literacy can exacerbate health inequalities by further widening the digital divide. From a health equity perspective, this pattern is consistent with the Inverse Care Law, which suggests that those with the greatest health needs often have the least access to effective healthcare resources (23). In the digital era, unequal access to digital technologies and differences in digital competencies may further reinforce this imbalance. Consequently, the health benefits of digital empowerment may exhibit a “Matthew effect,” whereby socioeconomically advantaged individuals are better positioned to acquire, understand, and use digital health information, while disadvantaged groups fall further behind, thereby widening existing health disparities (24). The 55th Internet Development Status Statistics Report of China shows that there is a significant disparity in internet access between urban and rural areas. The internet penetration rate in rural areas is 19.7 percentage points lower than in urban areas. This urban–rural digital divide places rural adults at a disadvantage regarding access to digital technology and proficiency in its utilization (25). In a longitudinal study found that rural adults in China are significantly less likely than their urban counterparts to use digital technology to improve their health (26). Structural disadvantages in offline society often extend into the digital sphere, limiting the ability of middle-aged and older rural adults to acquire, manage, and apply health information through digital tools (27), thereby perpetuating their disadvantage in digital health literacy (28). A recent empirical study indicates that rural adults in China not only have limited capacity to collect online health information but also demonstrate low engagement in digital health-related activities (29). Due to the lack of a supportive digital environment and necessary technical competencies, rural adults struggle to effectively navigate internet tools and smart devices to enhance their health, leading to widening disparities in health management between them and urban populations (30). Furthermore, digital health technologies tend to disproportionately benefit individuals with higher socioeconomic status (31). The persistence of structural inequalities, such as urban–rural and social class disparities, within the digital space creates a cumulative effect, ultimately exacerbating health disparities. Overall, the relationship between digital health literacy and urban–rural health disparities remains inconclusive, and existing literature provides limited discussion on this topic.

This study focuses on middle-aged and older adults aged 45 years and above, utilizing data from the 2021 Chinese General Social Survey (CGSS) to examine the impact of digital health literacy on urban–rural health disparities. Additionally, it analyzes variations in this impact across different demographic groups and explores the underlying mechanisms through pathway analysis. The findings of this study provide valuable policy implications for narrowing urban–rural health disparities, reducing health inequalities, and promoting digital health literacy among rural middle-aged and older adults.

## 2 Methods

### 2.1 Data

**Figure 1:**
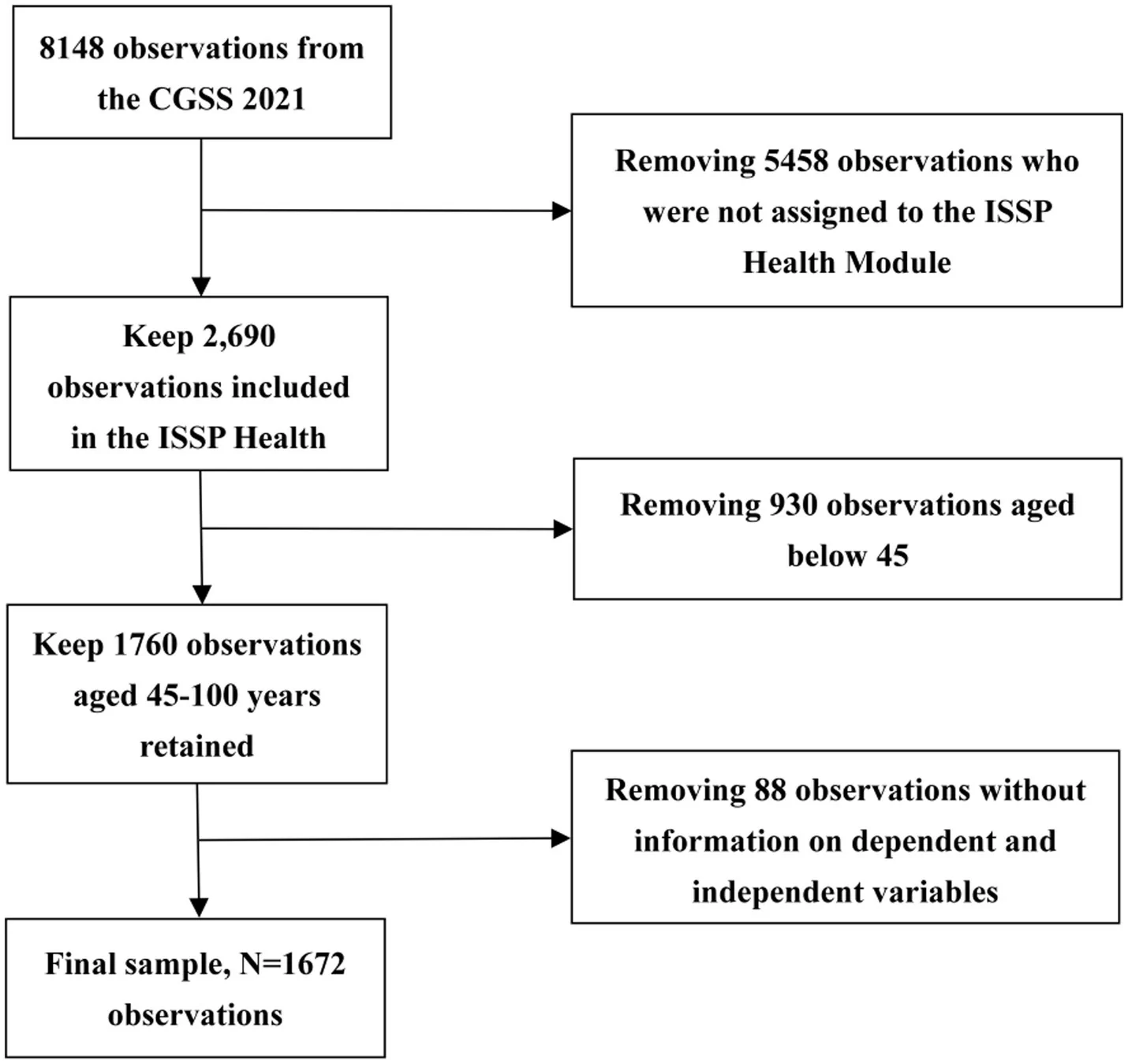
Flow diagram of the sample selection.

This study utilized data from the 2021 CGSS, a survey conducted by the Survey and Data Center of Renmin University of China. The CGSS employs a multistage, stratified probability proportional to size sampling design covering 21 provinces, autonomous regions, and municipalities across China, randomly selecting Chinese citizens aged 18 years and above to obtain a nationally representative sample. Through face-to-face interviews, the CGSS collects cross-sectional data on various aspects of society, communities, families, and individuals, and contains a total of 8,148 valid samples. The present study drew on the ISSP Health Module, which was administered to a randomly selected one-third subsample of CGSS respondents (N = 2,690). This module was selected because it includes the questions required to construct the measure of digital health literacy used in the analysis. Individuals younger than 45 years and respondents with missing values on key variables were excluded. After data cleaning, the final analytical sample consisted of 1,672 observations. The sample selection procedure is presented in Figure 1.

### 2.2 Variables

#### 2.2.1 Dependent variables

This study focused on health as the dependent variable. Health is a multidimensional and comprehensive construct, encompassing both physical and mental health. Following existing literature, physical health was assessed using the question: “How do you feel about your current state of physical health?” The responses ranged from 1 (very unhealthy) to 5 (very healthy) (32, 33). In this study, responses 1 (very unhealthy) and 2 (relatively unhealthy) were classified as “physically unhealthy” and assigned a value of 0, whereas responses 3 (average), 4 (relatively healthy), and 5 (very healthy) were classified as “physically healthy” and assigned a value of 1. Thus, the physical health variable was coded as 0 (unhealthy) and 1 (healthy). Mental health was measured using the question: “How often have you felt unhappy and depressed in the past four weeks?” The responses were: “Never = 5,” “Rarely = 4,” “Sometimes = 3,” “Frequently = 2,” and “Very frequently = 1.” In this study, individuals who responded with 3 (sometimes), 4 (rarely), or 5 (never) were classified as “mentally healthy” and assigned a value of 1, while individuals who responded with 1 (very frequently) or 2 (frequently) were classified as “mentally unhealthy” and assigned a value of 0. Consequently, the mental health variable was coded as 0 (unhealthy) and 1 (healthy).

#### 2.2.2 Independent variables

##### 2.2.2.1 Digital health literacy

One of the independent variables in this study was digital health literacy. To assess digital health literacy among the participants, this study constructed a composite index based on measurement dimensions adopted in previous research on digital health literacy (12). Existing studies have conceptualized digital health literacy as involving individuals’ ability to access, understand, evaluate, and utilize digital health information (9, 34). Accordingly, this study measured digital health literacy using three dimensions: “Internet Health Information Search Frequency,” “Perceived Impact of Internet Use on Health,” and “Evaluation of Internet Information Reliability.” “Internet Health Information Search Frequency” was measured using the following questions: “In the past 12 months, have you frequently searched the internet for information about healthy lifestyles?,” “In the past 12 months, have you frequently searched the internet for information related to anxiety, stress, etc.?,” and “In the past 12 months, have you frequently searched the internet for information about vaccination?” Each of these three questions originally had five response options, recorded as: “Never = 1,” “Rarely = 2,” “Sometimes = 3,” “Often = 4,” and “Always = 5.” “Perceived Impact of Internet Use on Health” was assessed based on responses to questions evaluating how internet information influences personal health behaviors and medical understanding: “In the past 12 months, do you agree that internet information has had a positive impact on your health behaviors?” and “In the past 12 months, do you agree that internet information has helped you understand what your doctor told you?” These two questions had five response options, recorded as: “Strongly agree = 1,” “Agree = 2,” “Neither agree nor disagree = 3,” “Disagree = 4,” and “Strongly disagree = 5.” The “Evaluation of Internet Information Reliability” measurement assessed participants’ ability to use the internet to determine the necessity of seeking medical attention and to evaluate the accuracy of such judgments. This was measured using the following questions: “Do you agree that the internet can help people assess the need to see a doctor?,” “Do you agree that the internet helps verify the appropriateness of doctors’ advice?,” and “Do you agree that it is difficult to determine the reliability of online health information?” The responses to these three questions were recorded as: “Strongly agree = 1,” “Agree = 2,” “Neither agree nor disagree = 3,” “Disagree = 4,” and “Strongly disagree = 5.” Finally, the entropy weight method was used to calculate the relative weights of the eight indicators and aggregate them into a composite digital health literacy index. This approach allows for an objective weighting of indicators and has been applied in previous empirical studies using large-scale survey data to measure digital health-related capabilities. The digital health literacy score ranged from 0 to 1, where higher values indicated greater levels of digital health literacy.

The entropy weight method was applied to objectively assign weights to the eight indicators and calculate the digital health literacy index. The calculation procedure consists of the following steps, with Equations (1–8) presenting the detailed calculation process:

(1) Dimensionless treatment of indicators. To standardize the positive and negative indicators, the range standardization method was used:

For positive indicators:


$$Z_{\mathrm{ij}}=(\chi_{\mathrm{ij}}-\chi_{\min})/(\chi_{\max}-\chi_{\min})$$ (1)

For negative indicators:


$$Z_{\mathrm{ij}}=(\chi_{\max}-\chi_{\mathrm{ij}})/(\chi_{\max}-\chi_{\min})$$ (2)

In formula (1) and (2): $\chi_{\mathrm{ij}}$ represent s j indicator of i individual; $\chi_{\max}$ and $\chi_{\min}$ were the maximum and minimum of j indicator among all evaluation objects; $Z_{\mathrm{ij}}$ was the dimensionless indicator value.

(2) Normalization of indicators.

$$P_{\mathrm{ij}}=Z_{\mathrm{ij}}/\sum_{i=1}^{m}Z_{\mathrm{ij}}$$ (3)

$P_{\mathrm{ij}}$ was the value of $Z_{\mathrm{ij}}$ after normalization treatment.

(3) Calculation of each indicator’s Entropy $E_{j}$

$$E_{j}=-k\sum_{i=1}^{m}Z_{\mathrm{ij}}$$ (4)

(4) Calculation of entropy redundancy $D_{j}$ .

$$D_{j}=1-E_{j}$$ (5)

(5) Calculation of each indicator’s objective weight $W_{j}$ .

$$W_{j}=D_{j}/\sum_{j=1}^{8}D_{j}$$ (6)

(6) Calculation of the digital health literacy index $Y_{i}$ .

$$Y_{i}=100\times \sum_{j=1}^{8}W_{j}\times Z_{\mathrm{ij}}$$ (7)

##### 2.2.2.2 Urban–rural

Another independent variable in this study was hukou status (urban or rural). In accordance with established methodologies, urban and rural classifications were based on household registration status (6). Individuals with non-agricultural household registration were categorized as urban (assigned a value of 1), while those with agricultural household registration were categorized as rural (assigned a value of 0).

#### 2.2.3 Control variables

Individual-level factors, such as age, gender, educational attainment, and ethnicity, may influence health outcomes (35). Therefore, this study incorporated the following control variables: age (continuous variable: 45–92 years); gender (dummy variable: male = 1, female = 0); educational attainment (dummy variable: high school and above = 1, junior high school and below = 0); marital status (dummy variable: married = 1, unmarried = 0); ethnicity (dummy variable: Han nationality = 1, other ethnic groups = 0); and personal income (continuous variable). The precise definitions, assignments, and descriptive statistics of these variables are presented in Table 1.

**Table 1:** Descriptive statistics of the sample characteristics (*N* = 1,672).

Variables		Definition	Mean	SD	Min	Max
Dependent variables						
	Physical health	1 = Healthy; 0 = Unhealthy	0.739	0.439	0	1
	Mental health	1 = Mentally healthy; 0 = Mentally unhealthy	0.873	0.333	0	1
Key independent variables						
	Digital health literacy	Calculated using the entropy weight method	0.230	0.207	0	0.854
	Hukou	1 = Urban; 0 = Rural	0.271	0.445	0	1
Control variables						
	Gender	1 = Male; 0 = Female	0.439	0.496	0	1
	Ethnicity	1 = Han; 0 = Other ethnic groups	0.935	0.247	0	1
	Educational attainment	1 = Senior high school or above; 0 = Junior high school or below	0.242	0.428	0	1
	Age	Age of participants (years)	61.936	10.295	45	92
	Marital status	1 = Married (with spouse); 0 = Unmarried	0.779	0.415	0	1
	Personal income	Total annual personal income (ten thousand yuan)	3.536	25.852	0	999.1

### 2.3 Model

This study employed the Linear Probability Model to examine the effects of digital health literacy on health inequality among middle-aged and older adults in urban and rural areas. The model was specified as follows:


$$\begin{array}{l} \text{Healt}h_{i}=\partial +\beta_{1}\text{Digita}l_{i}+\beta_{2}\text{Huko}u_{i}+\beta_{3}\text{Digita}l_{i}\\ \times \text{Huko}u_{i}+\beta_{4}X_{i}+\varepsilon_{i} \end{array}$$ (8)

$\text{Healt}h_{i}$ represents the dependent variable, including physical health and mental health, which were assigned values of 1 for healthy and 0 for unhealthy. $\text{Digita}l_{i}$ represents the digital health literacy index, calculated using the entropy-weight method, and $\beta_{1}$ represents the impact coefficient of digital health literacy on the health of middle-aged and older adults. $\text{Huko}u_{i}$ is a dummy variable for urban–rural classification (urban = 1, rural = 0), and 2 represents the impact coefficient of urban–rural on health of middle-aged and older adults. $\text{Digita}l_{i}\times \text{Huko}u_{i}$ is the interaction term between digital health literacy and urban–rural classification, capturing the differential effects of digital health literacy on health inequality based on its significance and direction (positive or negative). of $\beta_{3}$ . $X_{i}$ represents a series of control variables, and $\varepsilon_{i}$ is the random disturbance term.

## 3 Results

### 3.1 Descriptive statistics

The descriptive statistical analysis for each variable is presented in Table 1. The mean represents the central tendency of the data, while the standard deviation (SD) reflects the degree of dispersion, indicating how much the dataset deviates from the mean. For the dependent variables, the physical and mental health conditions of middle-aged and older adults are, on average, relatively good. For the key independent variables, digital health literacy scores are relatively low, and a larger proportion of middle-aged and older adults have rural household registrations. Regarding the control variables, the educational attainment of middle-aged and older adults is generally above the high school level; the mean personal income of middle-aged and older adults is 35,360 yuan, which is relatively low; and the sample includes a higher proportion of married, male, and Han individuals among middle-aged and older adults. In terms of variable dispersion, personal income exhibits the greatest variability, while digital health literacy scores show the least dispersion.

### 3.2 Main results

Table 2 presents the regression results examining the impact of digital health literacy, urban–rural classification, and their interaction term on the physical and mental health of middle-aged and older adults. After controlling for other variables, columns (1) and (3) report the effects of digital health literacy and urban–rural classification on physical and mental health, respectively, while columns (2) and (4) report the results including the interaction term between digital health literacy and urban–rural classification. Regarding physical health, column (1) shows that, on average, a one-unit increase in digital health literacy improves physical health by 0.2992 points [95% Confidence interval (CI) (0.1937, 0.4046), *p* = 0.000], and the physical health of urban middle-aged and older adults is 0.0706 points higher than that of their rural counterparts [95% CI (0.0254, 0.1159), *p* = 0.002]. Column (2) reveals that digital health literacy reduces physical health inequality among urban and rural adults by 0.2229 points [95% CI (−0.4329, −0.0129), *p* = 0.037], indicating that digital health literacy can help mitigate physical health disparities. Regarding mental health, column (3) indicates that a one-unit increase in digital health literacy improves mental health by 0.1089 points [95% CI (0.0262, 0.1916), *p* = 0.010], and the mental health of urban middle-aged and older adults is 0.0667 points higher than that of their rural counterparts [95% CI (0.0361, 0.0973), *p* = 0.000]. Column (4) shows that digital health literacy reduces mental health inequality between urban and rural middle-aged and older adults by 0.2410 points [95% CI (−0.3908, −0.0913), *p* = 0.002], slightly exceeding its effect on physical health, further confirming that digital health literacy plays a crucial role in mitigating mental health disparities. Overall, the results suggest that digital health literacy contributes to reducing health inequalities among middle-aged and older adults.

**Table 2:** Effect of digital health literacy on physical and mental health and urban–rural health inequalities.

Variables	Physical health		Mental health	
	(1)	(2)	(3)	(4)
Hukou (ref. = Rural)	0.0706***	0.1301***	0.0667***	0.1310***
	(0.0231)	(0.0410)	(0.0156)	(0.0262)
Digital health literacy score	0.2992***	0.3622***	0.1089***	0.1770***
	(0.0538)	(0.0616)	(0.0422)	(0.0510)
Hukou × Digital health literacy score		−0.2229**		−0.2410***
		(0.1071)		(0.0764)
Gender (ref. =Female)	0.0488**	0.0466**	0.0479***	0.0454***
	(0.0209)	(0.0209)	(0.0159)	(0.0159)
Age	−0.0044***	−0.0044***	0.0006	0.0007
	(0.0011)	(0.0011)	(0.0008)	(0.0008)
Ethnicity (ref. = Other ethnic groups)	0.0327	0.0326	0.0590	0.0588
	(0.0431)	(0.0433)	(0.0375)	(0.0375)
Personal income	−0.0001	−0.0001	−0.0006***	−0.0005**
	(0.0003)	(0.0003)	(0.0002)	(0.0002)
Educational attainment (ref. = Junior high school or below)	0.0780***	0.0817***	0.0411**	0.0451***
	(0.0232)	(0.0232)	(0.0162)	(0.0162)
Marital status (ref. = Unmarried)	0.0509*	0.0521*	0.0719***	0.0732***
	(0.0277)	(0.0277)	(0.0226)	(0.0225)
Constant	0.8152***	0.8005***	0.6518***	0.6359***
	(0.0890)	(0.0895)	(0.0706)	(0.0711)
Number of observations	1,672	1,672	1,672	1,672
R-squared	0.0786	0.0808	0.0459	0.0502

Standard errors in parentheses. “Ref.” refers to the reference group. *p < 0.1; **p < 0.05; ***p < 0.01.

Regarding control variables, several individual-level factors were significantly associated with health outcomes. Specifically, men exhibited better physical and mental health than women, married individuals showed better physical and mental health than unmarried individuals, higher education levels were positively associated with better health outcomes, and advanced age was associated with poorer physical health. Interestingly, higher income was negatively associated with mental health among middle-aged and older adults. One possible explanation is that higher-income individuals may experience greater psychological expectations and social comparison pressure (36), which could offset the potential mental health benefits associated with economic advantages.

### 3.3 Robustness checks

To verify the robustness of the previous estimation results, this study employs the Principal Component Analysis Method to compute the digital health literacy index, replacing the previously used Entropy Weight Method for re-estimation. The regression results using this alternative measurement are presented in Table 3, Columns (1)–(2). The findings indicate that the estimated coefficients of digital health literacy on physical and mental health remain significantly positive; the physical and mental health of urban middle-aged and older adults is significantly better than that of their rural counterparts; and the estimated coefficients of digital health literacy’s effect on urban–rural health inequalities are significantly negative at the 5% level, confirming that digital health literacy mitigates health disparities among middle-aged and older adults. Furthermore, this study conducts a 5% winsorization of the original digital health literacy index to reduce the influence of outliers on regression results. As shown in Table 3, Columns (3)–(4), middle-aged and older adults with higher digital health literacy and those residing in urban areas exhibit better physical and mental health, and digital health literacy significantly alleviates urban–rural disparities in physical and mental health. Notably, despite the changes in measurement methods and the adjustment for extreme values, the regression results remain consistent with the earlier findings, reinforcing the robustness of this study’s conclusions.

**Table 3:** Robustness checks of digital health literacy on physical and mental health.

Method	Replace key variable		Winsorization (5%)	
Dependent variables	Physical health	Mental health	Physical health	Mental health
	(1)	(2)	(3)	(4)
Hukou (ref. = Rural)	0.0792***	0.0758***	0.1242***	0.1251***
	(0.0244)	(0.0159)	(0.0422)	(0.0266)
Digital health literacy score	0.0482***	0.0236***	0.3815***	0.2010***
	(0.0083)	(0.0069)	(0.0738)	(0.0590)
Hukou × Digital health literacy score	−0.0299**	−0.0327***	−0.2022*	−0.2154***
	(0.0145)	(0.0103)	(0.1200)	(0.0809)
Constant	0.8852***	0.6776***	0.7989***	0.6275***
	(0.0852)	(0.0677)	(0.0924)	(0.0725)
Control variables included	YES	YES	YES	YES
Number of observations.	1,672	1,672	1,600	1,600
R-squared	0.0803	0.0502	0.0774	0.0520

Standard errors in parentheses. “Ref.” refers to the reference group. *p < 0.1; **p < 0.05; ***p < 0.01.

**Figure 2:**
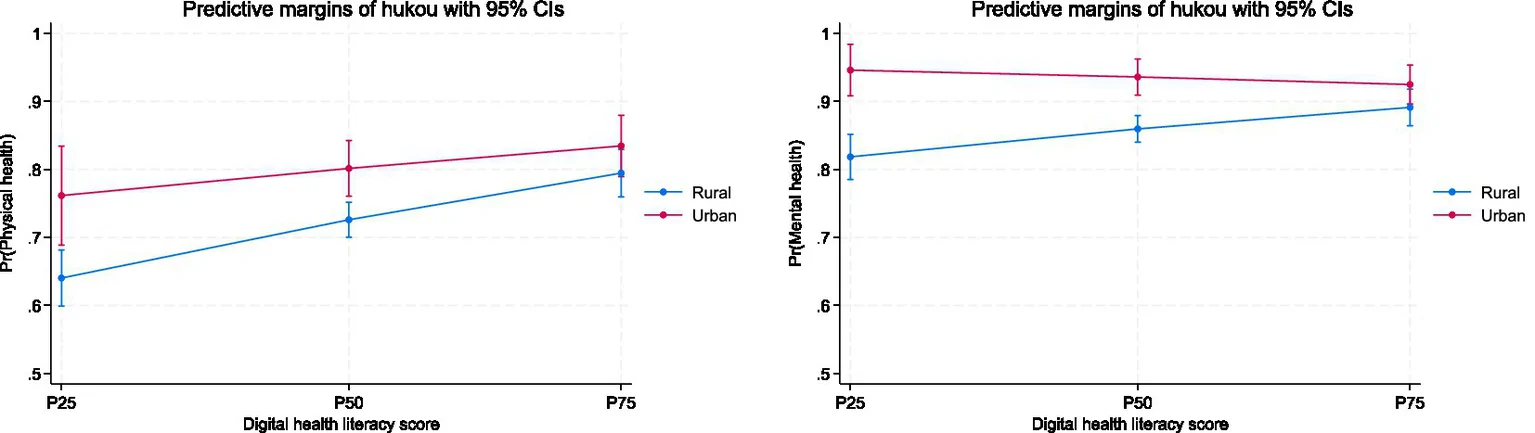
Predictive margins of hukou status by digital health literacy.

Considering that the dependent variable is binary, we further employ a Logit model as an alternative specification to examine the robustness of the baseline results. Since the coefficients of interaction terms in nonlinear models cannot be directly interpreted, we calculate predictive margins based on the Logit estimates and visualize the results in Figure 2. The figure shows that rural residents have lower predicted probabilities of good health than urban residents at relatively low levels of digital health literacy. However, the gap between rural and urban residents gradually narrows as digital health literacy increases. These findings are consistent with the baseline results and suggest that improving digital health literacy may help reduce health disparities between rural and urban populations.

### 3.4 Heterogeneity analysis

This study examines the heterogeneous effects of digital health literacy on the physical and mental health of middle-aged and older adults in urban and rural areas across three dimensions: marital status, educational attainment, and age.

#### 3.4.1 Marital status

Table 4, Columns (1)–(2), and Table 5, Columns (1)–(2), show that digital health literacy has a stronger positive effect on reducing physical and mental health inequalities among married middle-aged and older adults in urban and rural areas. Specifically, digital health literacy reduces physical health disparities between urban and rural middle-aged and older adults by 0.2584 points (95% CI [−0.4965, −0.0202], *p* = 0.033) and alleviates mental health inequalities by 0.2513 points (95% CI [−0.4106, −0.0920], *p* = 0.002). Conversely, no significant effect is observed on physical and mental health inequalities among unmarried middle-aged and older adults in urban and rural areas.

**Table 4:** Heterogeneity analysis – effect of digital health literacy on physical health.

Method	Marital status		Education level		Age	
Group	Others	Married	Low education	High education	Middle-aged	Older adults
	(1)	(2)	(3)	(4)	(5)	(6)
Hukou (ref. = Rural)	0.1326*	0.1086**	0.1343***	0.0228	0.1397*	0.1100**
	(0.0787)	(0.0484)	(0.0490)	(0.0803)	(0.0724)	(0.0505)
Digital health literacy score	0.1327	0.3832***	0.3570***	0.2689**	0.3555***	0.3145***
	(0.1730)	(0.0651)	(0.0739)	(0.1149)	(0.0788)	(0.1073)
Hukou × Digital health literacy score	0.0901	−0.2584**	−0.1093	−0.1105	−0.2659	−0.1267
	(0.2523)	(0.1214)	(0.1420)	(0.1792)	(0.1716)	(0.1581)
Constant	0.5232***	0.9143***	0.8352***	0.8120***	0.6395***	0.5016***
	(0.1826)	(0.0935)	(0.1084)	(0.1489)	(0.1743)	(0.1885)
Control variables included	Yes	Yes	Yes	Yes	Yes	Yes
Number of observations	369	1,303	1,265	403	785	887
R-squared	0.0605	0.0944	0.0614	0.0507	0.0745	0.0577

Standard errors in parentheses. “Ref.” refers to the reference group. *p < 0.1; **p < 0.05; ***p < 0.01.

**Table 5:** Heterogeneity analysis: the dependent variables is mental health.

Method	Marital status		Education level		Age	
Group	Others	Married	Low education	High education	Middle-aged	Older adults
	(1)	(2)	(3)	(4)	(5)	(6)
Hukou (ref. = Rural)	0.1339**	0.1237***	0.1453***	0.0309	0.1109**	0.1386***
	(0.0582)	(0.0280)	(0.0319)	(0.0489)	(0.0520)	(0.0311)
Digital health literacy score	0.1711	0.1666***	0.1923***	0.0417	0.0806	0.2617***
	(0.1437)	(0.0536)	(0.0604)	(0.0961)	(0.0674)	(0.0827)
Hukou × Digital health literacy score	−0.1740	−0.2513***	−0.2214**	−0.0752	−0.2069	−0.2814**
	(0.2040)	(0.0812)	(0.1107)	(0.1218)	(0.1329)	(0.1098)
Constant	0.4874***	0.7363***	0.6014***	0.8468***	0.6781***	0.3901***
	(0.1555)	(0.0723)	(0.0867)	(0.1080)	(0.1544)	(0.1395)
Control variables included	Yes	Yes	Yes	Yes	Yes	Yes
Number of observations	369	1,303	1,265	403	785	887
R-squared	0.0605	0.0944	0.0614	0.0507	0.0745	0.0577

Standard errors in parentheses. “Ref.” refers to the reference group. *p < 0.1; **p < 0.05; ***p < 0.01.

#### 3.4.2 Education attainment

To examine the heterogeneous effects of digital health literacy by educational attainment, the sample is divided into two groups: those with a high education level (high school and above) and those with a low education level (junior high school and below). As shown in Table 4, Columns (3)–(4), and Table 5, Columns (3)–(4), digital health literacy has a stronger effect on reducing mental health disparities among middle-aged and older adults with lower education levels, significantly reducing mental health inequalities among urban and rural middle-aged and older adults with low educational attainment by 0.2214 points [95% CI (−0.4386, −0.0042), *p* = 0.046]. However, digital health literacy does not significantly reduce physical health disparities among those with low educational attainment, nor does it significantly alleviate health inequalities among those with high educational attainment.

#### 3.4.3 Age

The sample is divided into high-age (60–92 years old) and low-age (45–59 years old) groups, using 60 years old as the cutoff point. As shown in Table 4, Columns (5)–(6), and Table 5, Columns (5)–(6), digital health literacy has a stronger effect on reducing mental health disparities among urban and rural high-age adults, significantly reducing mental health inequalities among urban and rural high-age adults by 0.2814 points (95% CI [−0.4970, −0.0659], *p* = 0.011). However, no significant effect is observed on physical health disparities among high-age adults, nor on physical and mental health disparities among low-age adults.

### 3.5 Mechanism analysis: health-promoting behaviors

Digital health literacy can directly influence health outcomes and can also indirectly enhance quality of life through health-promoting behaviors, such as physical exercise (37). Health-promoting behaviors may act as a moderating factor in the relationship between digital health literacy and health among middle-aged and older adults. This study measured health-promoting behaviors using the following two survey questions: “In the past year, did you often engage in physical exercise during your free time?” and “Do you often engage in physical exercise for at least 20 min, causing sweating or faster breathing?” Participants who answered “every day,” “several times a week,” or “several times a month” to both questions were categorized as “engaging in health-promoting behaviors” (coded as 1), while the remaining participants were categorized as “not engaging in health-promoting behaviors” (coded as 0).

As shown in Table 6, the coefficient of the interaction term between urban–rural classification and digital health literacy on health-promoting behaviors is −0.2274 [95% CI (−0.4531, −0.0016), *p* = 0.048], indicating that digital health literacy negatively moderates urban–rural disparities in health-promoting behaviors. This finding suggests that as digital health literacy improves, rural middle-aged and older adults increasingly use the internet to access fitness information and engage in home-based exercises, thereby increasing their participation in health-promoting behaviors. Consequently, health disparities between urban and rural middle-aged and older adults are gradually reduced. Therefore, health-promoting behaviors serve as a key mechanism through which digital health literacy helps mitigate urban–rural health inequalities among middle-aged and older adults.

**Table 6:** Mechanism analysis – effect of digital health literacy on health-promoting behaviors.

Variables	Health-promoting behaviors
Hukou (ref. = Rural)	0.0729*
	(0.0381)
Digital health literacy score	0.2577***
	(0.0653)
Hukou × Digital health literacy score	−0.2274**
	(0.1151)
Constant	−0.0081
	(0.0774)
Control variables included	YES
Number of observations	1,669
R-squared	0.0505

Standard errors in parentheses. “Ref.” refers to the reference group. *p < 0.1; **p < 0.05; ***p < 0.01.

## 4 Discussion

Health equity is a fundamental component of social justice, and the advancement of digital technologies presents new opportunities to address urban–rural health disparities. This study found that enhancing digital health literacy helps mitigate urban–rural health inequalities among middle-aged and older adults, reducing both physical and mental health disparities. These findings contribute to a deeper understanding of the relationship between digital health literacy, health inequalities, and urban–rural disparities, offering new perspectives for policy intervention design.

This study provides robust empirical evidence on the role of digital health literacy in reducing health inequalities between urban and rural middle-aged and older adults. It demonstrates that digital health literacy not only enhances physical and mental well-being but also plays a critical role in narrowing health disparities between urban and rural populations. While previous research has predominantly examined the direct health effects of digital health literacy (10, 38), these studies have largely overlooked its potential role in bridging urban–rural health inequities. Unlike prior studies that emphasize the digital divide (26, 29), this study highlights the inclusive potential of digital health literacy, revealing its effectiveness in reducing health disparities between urban and rural areas. Although the cross-sectional design limits the ability to establish definitive causal relationships, the theoretical framework of this study suggests that digital health literacy may serve as an important upstream capability in the digital era. By enabling individuals to access reliable health information, utilize digital health services, and engage in effective health management, digital health literacy may contribute to improved health behaviors and outcomes, particularly among middle-aged and older adults who face greater challenges in adapting to digital technologies. These findings underscore the importance of digital health literacy as an essential tool for health promotion and emphasize its potential as a key strategy for advancing health equity. In particular, in rural areas, where access to information technology remains limited, policy efforts should prioritize digital health education and the adoption of related technologies. Targeted initiatives should focus on improving digital health literacy among middle-aged and older adults in rural settings, thereby contributing to the reduction of health disparities.

This study also reveals heterogeneity in the impact of digital health literacy on urban–rural health inequalities across different subgroups. Digital health literacy exhibits stronger effects on reducing urban–rural disparities in physical and mental health among married individuals. This phenomenon may reflect the influence of marital status on health support, as married individuals tend to benefit from stronger social support networks, resource sharing, and joint health management advantages (39). In addition, spouses and adult children may provide digital assistance by helping older adults access, interpret, and utilize online health information and digital health services. Such spousal and intergenerational support may serve as a form of “digital proxy,” which strengthens the translation of digital health literacy into effective health management practices and health improvements. These factors may enhance their ability to leverage digital health literacy in mitigating urban–rural health disparities. Regarding mental health, the effect of digital health literacy on reducing urban–rural health disparities is particularly significant among individuals with low educational attainment and older age groups. Individuals with lower educational levels often face greater barriers to accessing health knowledge and resources (40), making digital health literacy an essential tool for improving their health outcomes. Similarly, older adults experience a higher burden of health issues and increased healthcare needs (41), and digital health literacy can help them navigate these challenges more effectively. Therefore, greater attention and resources should be directed toward enhancing digital health literacy among middle-aged and older adults with these characteristics. Policymakers designing digital health literacy initiatives should consider these subgroup-specific factors and implement targeted, personalized intervention strategies to maximize the effectiveness of these programs.

Furthermore, this study elucidates the mechanism through which digital health literacy reduces urban–rural health inequalities by promoting health behaviors. As digital health literacy improves, middle-aged and older adults demonstrate higher engagement in health-promoting behaviors, leading to improvements in both physical and mental well-being (42). Rural older adults often have limited access to exercise-related information, and digital health literacy provides them with scientific knowledge, techniques, and tools to engage in effective physical activity (43). These resources help them develop healthier lifestyle habits, enhance physical health, and reduce disease risk (15). This finding presents a new perspective, suggesting that policy efforts should prioritize enhancing digital health literacy, particularly by developing accessible digital exercise communities and interactive health platforms tailored for rural older adults. Such initiatives can encourage greater participation in health-promoting behaviors, improve overall health outcomes, and ultimately contribute to narrowing urban–rural health disparities.

Several limitations in this study should be acknowledged. First, this study employs a cross-sectional design, which precludes the establishment of causal relationships among the variables; therefore, future research should consider longitudinal or experimental studies to establish causal relationships more effectively. Second, although the digital health literacy index was constructed based on previous literature and existing empirical studies, it is not equivalent to internationally validated psychometric instruments, such as eHEALS. Moreover, the measurement relies on self-reported survey responses, which may introduce reporting bias. Future studies could further improve measurement validity by incorporating validated scales or objective assessments of digital health capabilities. Third, the mechanism analysis in this study focuses primarily on physical exercise as a representative health-promoting behavior. However, digital health literacy may influence health outcomes through multiple behavioral pathways, including healthcare utilization, medication adherence, and self-management practices. Future research could further explore these potential mechanisms to provide a more comprehensive understanding of how digital health literacy affects health outcomes.

## 5 Conclusion

Our findings confirm that digital health literacy not only enhances the physical and mental well-being of middle-aged and older adults but also effectively reduces urban–rural health inequalities. However, the impact of digital health literacy on urban–rural health disparities varies across different demographic groups. Regarding physical health, the mitigating effect of digital health literacy on urban–rural disparities is stronger among married individuals, while its impact on mental health disparities is more evident among married individuals, those with lower educational attainment, and older adults. Additionally, digital health literacy helps mitigate urban–rural health inequalities through health-promoting behaviors. Despite these benefits, a substantial digital health divide persists between urban and rural residents. Therefore, policy interventions should move beyond general digital health promotion and focus on targeted and accessible programs for rural middle-aged and older adults. Specifically, community-based digital health literacy training can be developed through village-level service platforms and grassroots healthcare centers, with training contents focusing on essential skills such as accessing reliable online health information, using digital healthcare services, and managing personal health information. Furthermore, digital health literacy education can be integrated into existing primary healthcare services, enabling community health workers to provide continuous guidance and support for older adults. These targeted measures may improve the ability of rural older adults to use digital health resources effectively and further narrow urban–rural health disparities.

## Data Availability

Publicly available datasets were analyzed in this study. This data can be found at: http://www.cnsda.org/index.php?r=projects/view&id=65635422.
